# Psychiatric comorbidities in women with cardiometabolic conditions with and without ADHD: a population-based study

**DOI:** 10.1186/s12916-023-03160-7

**Published:** 2023-11-20

**Authors:** Unnur Jakobsdottir Smari, Unnur Anna Valdimarsdottir, Thor Aspelund, Arna Hauksdottir, Edda Bjork Thordardottir, Catharina A. Hartman, Pontus Andell, Henrik Larsson, Helga Zoega

**Affiliations:** 1 Centre of Public Health Sciences, Faculty of Medicine, University of Iceland, Sturlugata 8, Reykjavík, 102 Iceland; 2 Unit of Integrative Epidemiology, Institute of Environmental Medicine, Karolinska Institutet, Stockholm, Sweden; 3 Department of Epidemiology, Harvard T.H. Chan School of Public Health, Boston, MA USA; 4 Mental Health Services, Landspitali, The National University Hospital of Iceland, Reykjavik, Iceland; 5 Interdisciplinary Center Psychopathology and Emotion Regulations (ICPE), Department of Psychiatry, University of Groningen, University Medical Center Groningen, Groningen, The Netherlands; 6 Unit of Cardiology, Department of Medicine, Karolinska Institutet, Heart and Vascular Division, Karolinska University Hospital, Stockholm, Sweden; 7 Department of Medical Epidemiology and Biostatistics, Karolinska Institutet, Stockholm, Sweden; 8 School of Medical Sciences, Örebro University, Örebro, Sweden; 9 School of Population Health, Faculty of Medicine and Health, UNSW Sydney, Sydney, NSW Australia

**Keywords:** Attention-deficit/hyperactivity disorder, Females, Cardiometabolic risk factors, Cardiovascular diseases, Type 2 diabetes, Hypertension, Obesity, Anxiety disorders, Mood disorders, Self-harm

## Abstract

**Background:**

Leveraging a large nationwide study of Icelandic women, we aimed to narrow the evidence gap around female attention-deficit/hyperactivity disorder (ADHD) and cardiometabolic comorbidities by determining the prevalence of obesity, hypertension, type 2 diabetes, and cardiovascular diseases among women with ADHD and examine the association between cardiometabolic conditions and co-occurring ADHD with anxiety and mood disorders, alcoholism/substance use disorder (SUD), self-harm, and suicide attempts.

**Methods:**

We conducted a cross-sectional analysis of the nationwide, all-female, population-based *SAGA Cohort Study* (*n* = 26,668). To ascertain diagnoses and symptoms, we used self-reported history of ADHD diagnoses, selected cardiometabolic conditions and psychiatric disorders, and measured current depressive, anxiety, and PTSD symptoms through appropriate questionnaires (PHQ-9, GAD-7, and PCL-5). We calculated age-adjusted prevalences of cardiometabolic conditions by women’s ADHD status and estimated adjusted prevalence ratios (PR) and 95% confidence intervals (CI), using modified Poisson regression models. Similarly, we assessed the association of cardiometabolic conditions and co-occurring ADHD with current psychiatric symptoms and psychiatric disorders, using adjusted PRs and 95% CIs.

**Results:**

We identified 2299 (8.6%) women with a history of ADHD diagnosis. The age-adjusted prevalence of having at least one cardiometabolic condition was higher among women with ADHD (49.5%) than those without (41.7%), (PR = 1.19, 95% CI 1.14–1.25), with higher prevalence of all measured cardiometabolic conditions (myocardial infarctions (PR = 2.53, 95% CI 1.83-–3.49), type 2 diabetes (PR = 2.08, 95% CI 1.66–2.61), hypertension (PR = 1.23, 95% CI 1.12–1.34), and obesity (PR = 1.18, 95% CI 1.11–1.25)). Women with cardiometabolic conditions and co-occurring ADHD had, compared with those without ADHD, substantially increased prevalence of (a) all measured mood and anxiety disorders, e.g., depression (PR = 2.38, 95% CI 2.19–2.58), bipolar disorder (PR = 4.81, 95% CI 3.65–6.35), posttraumatic stress disorder (PR = 2.78, 95% CI 2.52–3.07), social phobia (PR = 2.96, 95% CI 2.64–3.32); (b) moderate/severe depressive, anxiety, and PTSD symptoms with PR = 1.76 (95% CI 1.67–1.85), PR = 1.97 (95% CI 1.82–2.12), and PR = 2.01 (95% CI 1.88–2.15), respectively; (c) alcoholism/SUD, PR = 4.79 (95% CI 3.90–5.89); and (d) self-harm, PR = 1.47 (95% CI 1.29–1.67) and suicide attempts, PR = 2.37 (95% CI 2.05–2.73).

**Conclusions:**

ADHD is overrepresented among women with cardiometabolic conditions and contributes substantially to other psychiatric comorbidities among women with cardiometabolic conditions.

**Supplementary Information:**

The online version contains supplementary material available at 10.1186/s12916-023-03160-7.

## Background

Attention-deficit/hyperactivity disorder (ADHD) is an impairing neurodevelopmental disorder with prevalence of around 3–4% in adults [1, 2]. The persistence of ADHD symptoms from childhood to adulthood is substantial and around two thirds of children with ADHD have been estimated to have continued functional impairments as adults [3, 4]. Psychiatric comorbidities are common in ADHD, where people with ADHD have a decreased life expectancy and more than double the risk of premature death compared with individuals without ADHD [5, 6]. There is increasing recognition of a gender difference in symptoms and comorbidities in ADHD, but until recently, studies have primarily focused on male symptomatology [7, 8]. Internalizing symptoms of ADHD are more common in women than men, including higher prevalence of mood and anxiety comorbidities [9, 10]. Studies on adolescents also indicate higher risk of self-harm in females with ADHD compared to males, as well as compared to other females without ADHD [11, 12], but further research is needed. Moreover, both ADHD and self-harm are associated with suicide attempts, placing females with ADHD at risk of attempted or completed suicide [13–15]. However, further research on the female symptomatology of ADHD is needed [7].

Although much less studied than mood and anxiety disorders, emerging evidence suggests substantial comorbidity between adult ADHD and chronic conditions such as obesity, type 2 diabetes, hypertension, hyperlipidemia, and cardiovascular diseases [10, 16–21]. If not identified and treated, these conditions—hereafter termed *cardiometabolic conditions—*can lead to a considerable health burden, societal costs, and premature mortality [22, 23]. Systematic reviews, meta-analyses, and cohort studies [24–26] have demonstrated positive associations of cardiometabolic conditions with a range of psychiatric disorders, including mood, anxiety, and stress-related disorders. Comorbid psychiatric disorders have been shown to be associated with increased mortality rates in people suffering from cardiometabolic conditions [27]. In these studies, associations appear to be even more distinct among women than men [24, 25]. Furthermore, cardiometabolic conditions have been found to be associated with suicide ideation and suicide attempts, especially among those suffering from comorbid depression [28, 29]. Co-occurring cardiometabolic conditions and psychiatric disorders increase the risk of premature death, highlighting the importance of appropriate management of these common, chronic conditions.

Comorbidity between ADHD and cardiometabolic conditions can be traced to both genetic factors and certain lifestyle-related risk factors, e.g., abnormal eating patterns, physical inactivity, sleep disruption, and high alcohol and nicotine use, all of which are common among adults with ADHD [30–34]. However, current understanding of underlying mechanisms and consequences of co-occurring ADHD and cardiometabolic conditions is limited, especially in females.

Leveraging a large nationwide study of Icelandic women, we aimed to narrow the evidence gap around female ADHD and cardiometabolic comorbidity by determining the prevalence of obesity, hypertension, type 2 diabetes, and cardiovascular diseases among women with ADHD. To enhance our understanding of mental health among women with cardiometabolic conditions and co-occurring ADHD, we examined associations with anxiety and mood disorders, alcoholism and substance use disorder (SUD), self-harm, and suicide attempts.

## Methods

### Study setting and population

We conducted a cross-sectional analysis of the nationwide, population-based, all-female *Stress-And-Gene-Analysis (SAGA) Cohort Study*. The *SAGA Cohort Study* is a prospective cohort study launched in Iceland, in 2018. All women aged 18–69 years, residing in Iceland in March 2018 (*N* = 104,197), were invited to respond to an electronic questionnaire about their history of trauma and physical and mental health. To participate, women had to understand and be capable of responding in Icelandic and have access to electronic identification. In total, 30,403 women consented to participate in the SAGA Cohort study. The distribution of socio-demographic factors of the SAGA cohort participants is comparable with the Icelandic female population [35, 36].

### Study measures

#### Ascertainment of ADHD and cardiometabolic conditions

To ascertain women’s ADHD, we used self-reported lifetime history of an ADHD diagnosis (“Have you *ever* been diagnosed with attention-deficit/hyperactivity disorder or primarily inattentive ADHD?”).

We also used self-reported lifetime diagnoses to ascertain the following cardiometabolic conditions: hypertension, type 2 diabetes, and cardiovascular disease (myocardial infarction), and obesity (BMI ≥ 30) was ascertained based on current self-reported height and weight. We created a composite variable “*any cardiometabolic condition*” as having history of one or more of these above mentioned cardiometabolic conditions.

#### Ascertainment of psychiatric disorders and symptoms

To ascertain relevant psychiatric disorders, we used self-reported lifetime history of diagnoses of mood and anxiety disorders, and alcoholism or substance use disorders (SUD). In this study, we considered mood disorders (major depression, bipolar disorder), anxiety and stress-related disorders (social phobia, generalized anxiety disorder (GAD), obsessive–compulsive disorder (OCD), posttraumatic stress disorder (PTSD)), and mental and behavioral disorders due to psychoactive substance (alcoholism, substance use disorder (SUD)).

We additionally assessed current symptoms (over the past 2 weeks) of depression with the *Patient Health Questionnaire-9* (PHQ-9) [37] and anxiety with the *General Anxiety Disorder-7* (GAD-7) [38]. To define moderate to severe symptoms of depression and anxiety, we used a cut-off score of 10 [37, 38]. We used the *PTSD Checklist for DSM-5* (PCL-5) to assess current symptoms (over the past month) of post-traumatic stress disorder (PTDS) with a cut-off score of 33 [39].

We ascertained lifetime history of self-harm and suicide attempts in all respondents with questions from the *World Health Organization (WHO) Composite International Diagnostic Interview* (CIDI) [40].

#### Socio-demographic factors

Demographic characteristics included the following: age (mean age, 18–29, 30–39, 40–49, 50–59, and 60–69 years), relationship status (married or in a relationship, single), number of children (0, 1–2, 3 +), education level (highest education level: primary, secondary education (high school or vocational education), college or equivalent (BSc or equivalent), and postgraduate (MSc or above)), monthly income (in Euros: < 981, 981–1960, 1961–3264, 3265–4559, > 4560 (low, low-medium, medium, high-medium, high income, respectively), conversion rates according to Central Bank of Iceland, February 15, 2023), employment status (employed/studying, retired/disability/sick leave), disability benefits (no, yes), smoking status (never, former or current), current other nicotine use (no, yes), binge drinking (defined as drinking 6 or more alcoholic drinks monthly or more often, less than once per month, or never, over the past 12 months) (Table 1).

**Table 1:** Characteristics of women with and without ADHD, number (%) or mean ± standard deviation

	**Non-ADHD** **(*N* = 24,369)**	**ADHD** **(*N* = 2299)**
**Age**		
Mean (SD)	44.9 (13.5)	36.7 (12.5)
**Age group, years**		
18–29	4090 (16.8%)	836 (36.4%)
30–39	4754 (19.5%)	580 (25.2%)
40–49	5531 (22.7%)	454 (19.7%)
50–59	5888 (24.2%)	302 (13.1%)
60–69	4106 (16.8%)	1127 (5.5%)
**Relationship status**		
In a relationship	18,647 (76.5%)	1503 (65.4%)
Single	5607 (23.0%)	773 (33.6%)
Unknown	115 (0.5%)	23 (1.0%)
**Number of children**		
0	4408 (18.1%)	697 (30.3%)
1–2	9632 (39.5%)	845 (36.8%)
3 +	10,192 (41.8%)	739 (32.1%)
Unknown	137 (0.6%)	18 (0.8%)
**Education**		
Elementary	3255 (13.4%)	601 (26.1%)
High school	7310 (30.0%)	857 (37.3%)
University	7933 (32.6%)	550 (23.9%)
University, higher education	5787 (23.7%)	275 (12.0%)
Unknown	84 (0.3%)	16 (0.7%)
**Monthly income, in Euros**		
Low (< 980)	1553 (6.4%)	252 (11.0%)
Low-medium (981–1960)	5234 (21.5%)	828 (36.0%)
Medium (1961–3264)	7307 (30.0%)	704 (30.6%)
High-medium (3265–4559)	5843 (24.0%)	302 (13.1%)
High (> 4560)	3493 (14.3%)	129 (5.6%)
Unknown	939 (3.9%)	84 (3.7%)
**Employment status**		
Employed/studying	20,723 (85.0%)	1725 (75.0%)
Retired/disability/sick leave	3491 (14.3%)	550 (23.9%)
Unknown	155 (0.6%)	24 (1.0%)
**Disability benefits**		
Yes	2145 (8.8%)	514 (22.4%)
No or unknown	22,224 (91.2%)	1785 (77.6%)
**Smoking status**		
Never	11,792 (48.4%)	776 (33.8%)
Former	8808 (36.1%)	907 (39.5%)
Current	3486 (14.3%)	588 (25.6%)
Unknown	283 (1.2%)	28 (1.2%)
**Other nicotine use**		
No	21,089 (86.5%)	1630 (70.9%)
Yes	3011 (12.4%)	635 (27.6%)
Unknown	269 (1.1%)	34 (1.5%)
**Binge drinking**		
Monthly or more often	3219 (13.2%)	442 (19.2%)
Never or less than once a month	20,849 (85.6%)	1831 (79.6%)
Unknown	301 (1.2%)	26 (1.1%)

*Abbreviations*: *ADHD* Attention-deficit/hyperactivity disorder, *CI* Confidence interval, *CMC* Cardiometabolic condition, *GAD* Generalized anxiety disorder, *OCD* Obsessive–compulsive disorder, *PTSD* Posttraumatic stress disorder, *SUD* Substance-use disorder

#### Data analysis

In total, 30,403 women consented to participate in the SAGA Cohort study. We excluded participants who did not respond to all baseline questions on medical history (*n* = 3715) as ascertainment of ADHD and cardiometabolic conditions was not possible without this information (see flowchart Additional file 1: Fig. S1). The cohort available for analysis thus comprised of 26,668 women.

First, we described women’s sociodemographic characteristics and health behaviors according to women’s diagnosis of ADHD by calculating frequencies and measures of central tendency and dispersion. We then estimated the age-adjusted prevalence of each cardiometabolic condition separately (obesity, hypertension, type 2 diabetes, myocardial infarction) and overall (any cardiometabolic condition) by women’s ADHD diagnosis, using logistic regression models. We assessed associations between ADHD and cardiometabolic conditions by calculating age-adjusted prevalence ratios (PR) and accompanying 95% confidence intervals (CI) separately for each measured cardiometabolic condition and overall, using modified Poisson regression models. We present estimates stratified by women’s age (18–30, 31–45, and 46–69 years) as additional files (Additional files 2, 3 and 4: Fig. S2-S4). Age was measured at the time when the women responded to the survey, not at the time of their diagnoses. We explored the contribution of the women’s education level, monthly income, and marital status on the observed associations by calculating PRs and accompanying 95% CIs for each measured cardiometabolic condition and overall, adjusting for age, education level, monthly income, and marital status.

To evaluate associations of relevant psychiatric disorders with cardiometabolic conditions and co-occurring ADHD, we used modified Poisson regression models to estimate age-adjusted PRs and 95% CIs for lifetime history of any psychiatric disorder, each specific disorder (major depression, bipolar disorder, social phobia, GAD, PTSD, OCD and alcoholism or SUD) and current symptoms of depression, anxiety, and PTSD, and for lifetime history of self-harm or suicide attempts. We explored the contribution effects of the women’s education level, monthly income, and marital status (SES) on the observed associations by calculating PRs and accompanying 95% CIs for each measured cardiometabolic condition and overall, adjusting for age, education level, monthly income, and marital status.

Finally, we examined the number of lifetime psychiatric diagnoses among women with cardiometabolic conditions with and without co-occurring ADHD, by calculating the age-adjusted prevalence of having been diagnosed with one, two, three, four, or five or more, mood, anxiety, and SUD disorders, using stratified multinomial regression models with age as a covariate.

We used R (version 4.2.2) for all analyses. The SAGA Cohort Study was approved by the National Bioethics Committee of Iceland (no. VSNb2017110046/03.01) and the Icelandic Data Protection Authority. All participants gave informed consent before participation.

## Results

Among a total of 26,668 women, 2299 (8.6%) reported having an ADHD diagnosis (mean age 36.7 years, SD 12.5 years) and 10,957 (41.1%) reported any cardiometabolic condition (mean age of 47.6 years, SD 12.9 years). A total of 978 (3.7%) women reported having both ADHD and one or more cardiometabolic condition (mean age 39.6 years, SD 12.5 years).

Compared with those without ADHD, women with an ADHD diagnosis were younger (36.7 years vs. 44.9 years), more likely to be single (33.6% vs. 23.0%), more likely not to have children (30.3% vs. 18.1%), less likely to have finished higher education, i.e., university and university higher education (35.9% vs. 56.3%), more likely to have low or low-medium income (47.0% vs. 27.9%), and more likely to be inactive from work (retired/disability/sick leave) (23.9% vs. 14.3%)―particularly because of a disability (22.4% vs. 8.8% on disability benefits). They were also more likely to smoke (25.6% vs. 14.3%), use other nicotine (27.6% vs. 12.4%), and binge drink monthly or more often (19.2% vs. 13.2%) (Table 1).

### ADHD and prevalence of cardiometabolic conditions

**Fig. 1:**
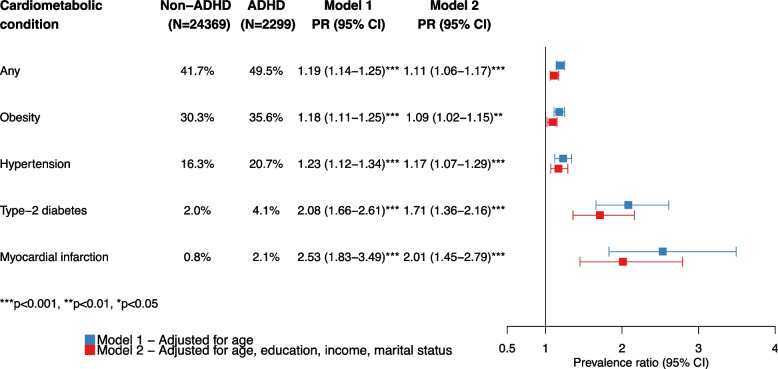
Age-adjusted prevalence and prevalence ratios of cardiometabolic conditions among women with and without ADHD. *Abbreviations*: ADHD, attention-deficit/hyperactivity disorder; CI, confidence interval; CMC, cardiometabolic condition; GAD, generalized anxiety disorder; OCD, obsessive–compulsive disorder; PTSD, posttraumatic stress disorder; SUD, substance-use disorder

Adjusting for age, the prevalence of having at least one of the assessed cardiometabolic conditions was higher among women with ADHD (49.5%) than among those without ADHD (41.7%), yielding an age-adjusted PR of 1.19 (95% CI 1.14–1.25) (Fig. 1). This association was evident across all age groups, with PRs of 1.30 (95% CI 1.17–1.45), 1.18 (95% CI 1.10–1.28), and 1.09 (95% CI 1.02–1.18) among women aged 18–30 years, 31–45 years, and 46–69 years, respectively (Additional file 2: Fig. S2).

Examining the prevalence of specific cardiometabolic conditions in association with ADHD, age-adjusted prevalence was higher for each condition in women with vs. without ADHD: obesity (35.6% vs. 30.3%), hypertension (20.7% vs. 16.3%), type 2 diabetes (4.1% vs. 2.0%), and myocardial infarction (2.1% vs. 0.8%). We observed the highest PRs for history of myocardial infarction (PR 2.53, 95% CI 1.83–3.49) and type 2 diabetes (PR 2.08, 95% CI 1.66–2.61), while the PRs were lower, but still significant, for hypertension (PR 1.23, 95% CI 1.12–1.34) and obesity (PR 1.18, 95% CI 1.11–1.25) (Fig. 1). In the youngest age group (18–30 years), we observed elevated PRs for type 2 diabetes (PR 4.17, 95% CI 1.73–10.00) and myocardial infarction (PR 3.30, 95% CI 1.55–7.02) (Additional file 3: Fig. S3). Adjusting for education, monthly income and marital status attenuated these PRs only slightly, suggesting that the observed associations are robust across socio-demographic factors (Fig. 1).

### Anxiety and mood disorders and self-harm in women with ADHD and co-occurring cardiometabolic conditions

**Fig. 2:**
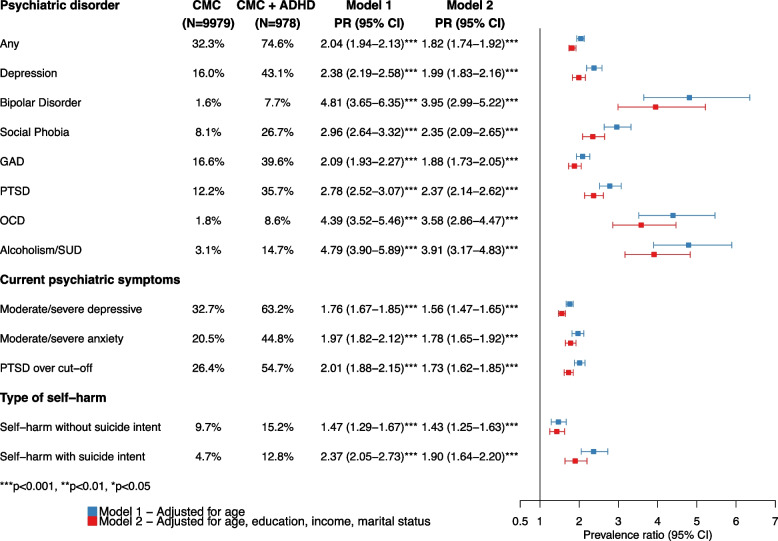
Age-adjusted prevalence and prevalence ratios of psychiatric disorders among women with cardiometabolic conditions and with/without co-occurring ADHD. *Abbreviations*: ADHD, attention-deficit/hyperactivity disorder; CI, confidence interval; CMC, cardiometabolic condition; GAD, generalized anxiety disorder; OCD, obsessive–compulsive disorder; PTSD, posttraumatic stress disorder; SUD, substance-use disorder

**Fig. 3:**
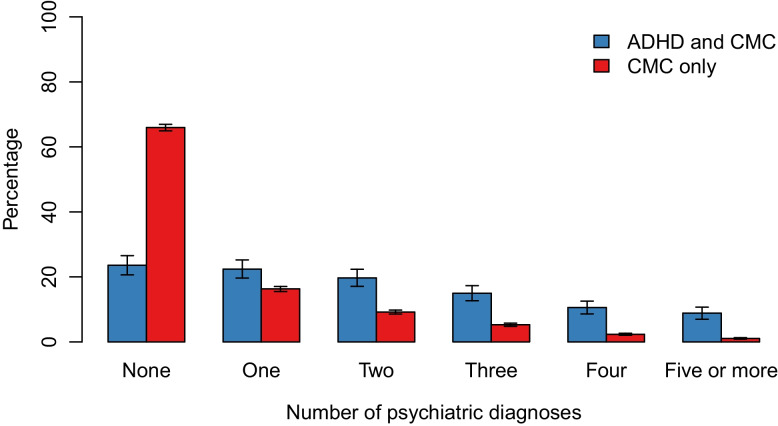
Number of psychiatric disorders among women with cardiometabolic conditions and with/without co-occurring ADHD. Prevalence is age-adjusted. *Abbreviations*: ADHD, attention-deficit/hyperactivity disorder; CI, confidence interval; CMC, cardiometabolic condition; GAD, generalized anxiety disorder; OCD, obsessive–compulsive disorder; PTSD, posttraumatic stress disorder; SUD, substance-use disorder

The prevalence of mood and anxiety disorders and alcoholism/SUD was higher among women with cardiometabolic conditions and co-occurring ADHD (74.6%) compared with those without co-occurring ADHD (32.3%), yielding an age-adjusted PR of 2.04 (95% CI 1.94–2.13). This association was evident for all measured psychiatric disorders, with age-adjusted PRs of 2.38 (95% CI 2.19–2.58) for depression, 4.81 (95% CI 3.65–6.35) for bipolar disorder, 2.96 (95% CI 2.64–3.32) for social phobia, 2.09 (95% CI 1.93–2.27) for GAD, 2.78 (95% CI 2.52–3.07) for PTSD, 4.39 (95% CI 3.52–5.46) for OCD, and 4.79 (95% CI 3.90–5.89) for alcoholism/SUD (Fig. 2). Among women with cardiometabolic conditions with co-occurring ADHD, over a third (34.3%) of the women had been diagnosed with three or more of the psychiatric disorders assessed, as compared with only 8.7% of those without co-occurring ADHD (Fig. 3). The prevalence of all measured psychiatric disorders was highest in the youngest age group (18–30 years) with 86.1% of women with cardiometabolic conditions and ADHD having been diagnosed with any of the measured psychiatric disorders, compared to 50.1% of the women with cardiometabolic conditions without ADHD (PR = 1.72, 95% CI 1.60–1.85). However, the difference between these groups was most evident in the oldest age group (46 years and older) with 72.6% of the women with cardiometabolic conditions and ADHD having been diagnosed with any of the measured psychiatric disorders, compared with 24.8% of the women with cardiometabolic conditions without ADHD, yielding a PR of 2.92 (95% CI 2.70–3.16) (Additional file 4: Fig. S4).

The prevalence of current moderate/severe depressive symptoms, moderate/severe anxiety symptoms, and PTSD symptoms was higher among those with cardiometabolic conditions and co-occurring ADHD compared with those without ADHD (63.2% vs. 32.7%, 44.8% vs. 20.5%, 54.7% vs. 26.4%, respectively), yielding age-adjusted PRs of 1.76 (95% CI 1.67–1.85), 1.97 (95% CI 1.82–2.12), and 2.01 (95% CI 1.88–2.15), respectively (Fig. 2). The prevalence of having history of self-harm or a suicide attempt was also higher among women with cardiometabolic conditions and co-occurring ADHD than among those without ADHD (15.2% vs. 9.7% and 12.8% vs. 4.7%, respectively), yielding age-adjusted PRs of 1.47 (95% CI 1.29–1.67) for self-harm and 2.37 (95% CI 2.05–2.73) for suicide attempts (Fig. 2). Adjusting for education, monthly income and marital status attenuated these PRs minimally, suggesting a minor influence of these factors on the observed associations (Fig. 2).

## Discussion

This nationwide population-based study on women with co-occurring ADHD and cardiometabolic conditions and their association with anxiety and mood disorders, alcoholism and substance use disorder (SUD), self-harm, and suicide attempts generated two main findings. First, cardiometabolic conditions, including obesity, hypertension, type 2 diabetes, and myocardial infarctions, were more prevalent in women with a diagnosis of ADHD than in those without. Second, women with cardiometabolic conditions and co-occurring ADHD had a 2- to 5-fold higher risk of all measured history of psychiatric diagnoses compared with women with cardiometabolic conditions without ADHD.

These findings align with emerging evidence showing an association between ADHD and cardiometabolic conditions [10, 17, 19]. The PRs of type 2 diabetes and myocardial infarctions were substantially higher in women with ADHD compared to those without ADHD (2.08 and 2.53, respectively). This was especially evident in the youngest age group (18–30 years) suggesting that women with ADHD may develop serious cardiometabolic conditions at an earlier age than those without ADHD. In line with previous findings [41, 42], our results show higher prevalence of lifestyle-related risk factors associated with cardiometabolic conditions among women with ADHD, including smoking and binge drinking, as well as lower education, emphasizing the need for preventive strategies, i.e., diagnosing ADHD early and providing educational support for girls and women with ADHD. The findings also have importance for clinicians treating women with ADHD. Clinicians need to be vigilant of cardiometabolic risk factors and the high risk of serious cardiometabolic conditions in women with ADHD. Findings have indicated that ADHD medication treatment may diminish the risk of some negative health behaviors and thereby lower the risk of poor somatic health outcomes [43], but further research is needed. ADHD medication can increase heart rate and blood pressure, raising concerns about their cardiovascular safety. However, a recent meta-analysis showed no significant association between ADHD medication and cardiovascular diseases across age-groups, though a modest increase in risk could not be ruled out [44].

Our findings indicate that women with cardiometabolic conditions and co-occurring ADHD have a notably elevated prevalence of poor mental health outcomes, e.g., increased anxiety and depressive symptoms. Poor mental health has been linked to worse outcomes in cardiovascular diseases [27]. The high prevalence rate of depressive symptoms is alarming with nearly two thirds of the women with ADHD and cardiometabolic conditions reporting moderate/severe depressive symptoms. Moreover, roughly half of these women have PTSD symptoms above the threshold score, and nearly half of them have moderate/severe anxiety symptoms. Studies have indicated that ADHD medication could reduce the risk of developing depression [45] emphasizing the importance of clinical management of ADHD. In our data, women with co-occurring ADHD and cardiometabolic conditions clearly had an elevated prevalence of psychiatric multimorbidity and history of self-harm and suicide attempts, putting them at risk for severely impaired quality of life and premature death. Clinicians treating women with cardiometabolic conditions and co-occurring ADHD need to be aware of the risk of poor mental health outcomes in this population and the potential impacts on disease management and prognosis. This patient group will benefit from a multi-disciplinary approach, incorporating psychiatrists, psychologists, and cardiologists.

## Limitations

A strength of this study is a relatively large sample size that is representative of the female adult population in Iceland regarding demographic characteristics. Yet, there are several limitations to be noted. First, this is a cross-sectional study, and we have no information on how or when the psychiatric disorders or cardiometabolic disorders were diagnosed. However, as ADHD typically arises early in development, it precedes the onset of most physical health conditions and mood and anxiety disorders [46, 47]. Second, as the *SAGA Cohort Study* is a study on trauma history, it could be that women who had experienced trauma were more likely to participate in the study than women who had not. These women could be more likely to suffer from psychiatric disorders than other women. For example, 8.6% of the women in our study reported a diagnosis of ADHD, which is higher than the reported prevalence of ADHD among female adults in the general population [2]. Third, diagnoses of cardiometabolic conditions, ADHD, and psychiatric disorders are self-reported which could lead to misclassification of the reported diagnoses; it is difficult to infer the implications of this for our findings as diagnoses can both be underreported and overreported. As we rely on information on history of diagnoses, only women who have been diagnosed with ADHD were included in the analyses, i.e., women with undiagnosed ADHD were not included in the analyses. The measured associations between ADHD and cardiometabolic conditions, and the association with psychiatric disorders might therefore be underestimated in the results as one could assume that women with undiagnosed ADHD are also at increased risk of cardiometabolic and psychiatric comorbidities. In future studies, it would be interesting to examine the same associations stratified by sex and age. Furthermore, it would be interesting to examine the role of lifestyle-related factors associated with ADHD on the development of cardiometabolic conditions and, in turn, the prognosis of psychiatric disorders. It would have important clinical implications to understand if providing behavioral support or treatment for relevant changes in lifestyle could hinder the development of these disorders. Finally, it would be interesting to examine the role of ADHD medication on the development of cardiometabolic conditions and psychiatric comorbidities to understand if medication could act as a protective factor or possibly as a risk factor for this association.

## Conclusions

Our findings highlight the role of ADHD in cardiometabolic conditions and associated psychiatric comorbidities. Our findings indicate that women with ADHD could develop these conditions at an earlier age than other women. Furthermore, women suffering from cardiometabolic conditions and co-occurring ADHD have increased prevalence of both mood and anxiety disorders as well as having a diagnosis of alcoholism or substance use disorder. Moreover, they are at an increased risk of self-harm and suicide attempts. Clinicians, in the field of psychiatry, cardiology, and endocrinology, need to be watchful of the psychiatric and cardiometabolic problems that commonly affect adult women with ADHD to increase their well-being and quality of life and to decrease the risk of early death. These findings also stress the importance of diagnosing women with ADHD early. Treating their symptoms and providing educational and behavioral support could reduce the likelihood of them developing cardiometabolic conditions and psychiatric disorders, by diminishing the impact of lifestyle-related risk factors for cardiometabolic conditions that are often associated with ADHD.

### Supplementary Information

**Additional file 1: Figure S1.** Flowchart of the study sample.

**Additional file 2: Figure S2.** Prevalence and prevalence ratios of any cardiometabolic condition among women with and without ADHD stratified by age. *Abbreviations*: ADHD, attention-deficit/hyperactivity disorder; CI, confidence interval; CMC, cardiometabolic condition; GAD, generalized anxiety disorder; OCD, obsessive-compulsive disorder; PTSD, posttraumatic stress disorder; SUD, substance-use disorder.

**Additional file 3: Figure S3.** Prevalence and prevalence ratios of cardiometabolic conditions among women with and without ADHD stratified by age. *Abbreviations*: ADHD, attention-deficit/hyperactivity disorder; CI, confidence interval; CMC, cardiometabolic condition; GAD, generalized anxiety disorder; OCD, obsessive-compulsive disorder; PTSD, posttraumatic stress disorder; SUD, substance-use disorder.

**Additional file 4: Figure S4.** Prevalence and prevalence ratios of psychiatric disorders among women with cardiometabolic conditions and with/without co-occurring ADHD stratified by age. *Abbreviations*: ADHD, attention-deficit/hyperactivity disorder; CI, confidence interval; CMC, cardiometabolic condition; GAD, generalized anxiety disorder; OCD, obsessive-compulsive disorder; PTSD, posttraumatic stress disorder; SUD, substance-use disorder.

## Data Availability

Data are stored in encrypted form in the data centers of the software company SMART-TRIAL and at the University of Iceland Computing Institute. The procedure was approved by the Icelandic Data Protection Authority. Data cannot be put into a public data repository due to regulations but can be accessed through applications. More information on data application can be found in the following link: https://afallasaga.is/.

## Abbreviations

ADHD: Attention-deficit/hyperactivity disorder
CI: Confidence interval
CMC: Cardiometabolic condition
GAD: Generalized anxiety disorder
OCD: Obsessive-compulsive disorder
PR: Prevalence ratio
PTSD: Posttraumatic stress disorder
SES: Socio-economic status
SD: Standard deviation
SUD: Substance-use disorder
