# Supplementary figures and images for: Psychiatric comorbidities in women with cardiometabolic conditions with and without ADHD: a population-based study
Source: BMC Med. 2023 Nov 20;21:450. doi: 10.1186/s12916-023-03160-7 (PMC10659052; doi:10.1186/s12916-023-03160-7)

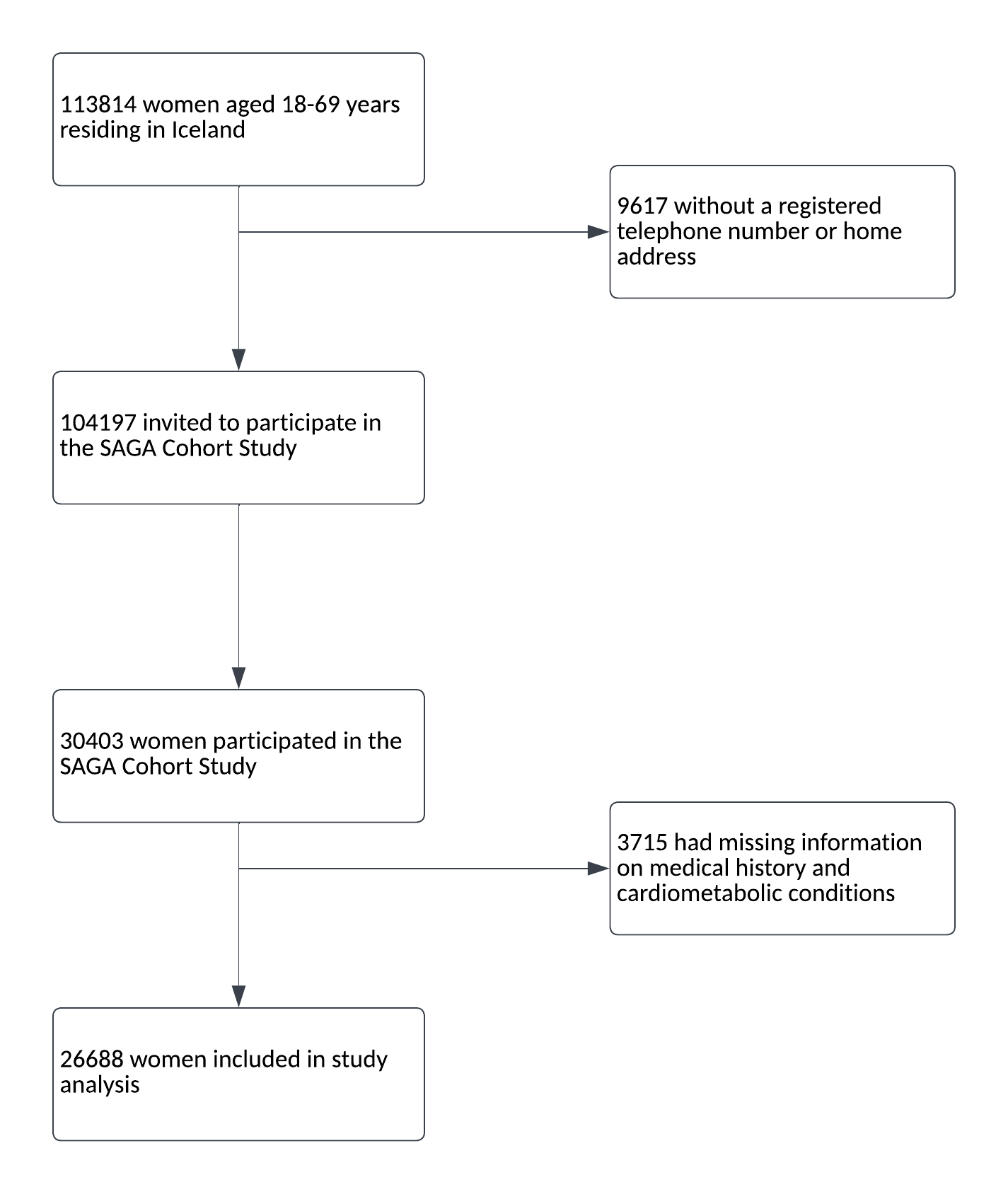

Supplement: Supplementary file 1 — Additional file 1: Figure S1. Flowchart of the study sample. [file 12916_2023_3160_MOESM1_ESM.png]

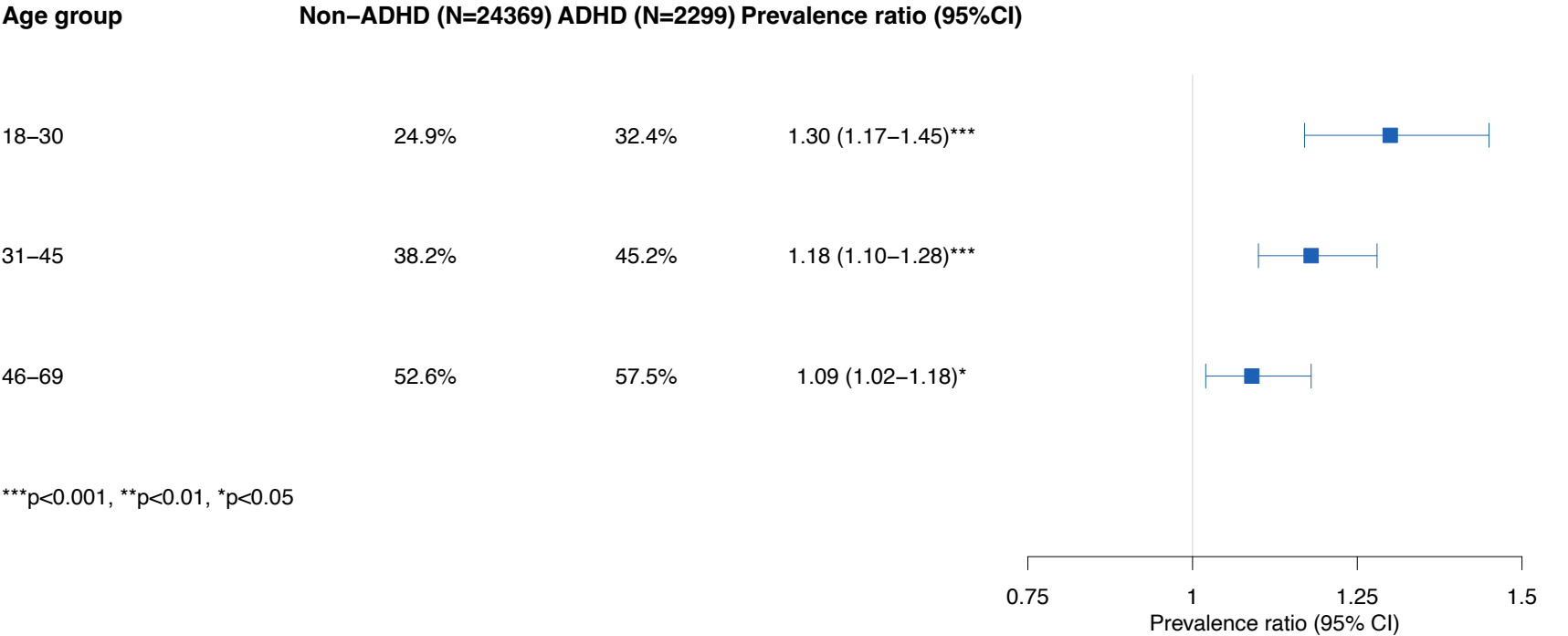

Supplement: Supplementary file 2 — Additional file 2: Figure S2. Prevalence and prevalence ratios of any cardiometabolic condition among women with and without ADHD stratified by age. Abbreviations: ADHD, attention-deficit/hyperactivity disorder; CI, confidence interval; CMC, cardiometabolic condition; GAD, generalized anxiety disorder; OCD, obsessive-compulsive disorder; PTSD, posttraumatic stress disorder; SUD, substance-use disorder. [file 12916_2023_3160_MOESM2_ESM.pdf]
