# Supplementary material for: Psychiatric comorbidities in women with cardiometabolic conditions with and without ADHD: a population-based study
Source: BMC Med. 2023 Nov 20;21:450. doi: 10.1186/s12916-023-03160-7 (PMC10659052; doi:10.1186/s12916-023-03160-7)

a) 18–30 years

Cardiometabolic condition	Non–ADHD (N=4564)	ADHD (N=895)	Prevalence ratio (95% CI)
Any	24.9%	32.4%	1.30 (1.17–1.45)***
Obesity	22.7%	28.3%	1.25 (1.11–1.40)***
Hypertension	3.2%	5.9%	1.83 (1.34–2.48)***
Type–2 diabetes	0.2%	1.0%	4.17 (1.73–10.00)**
Myocardial infarction	0.4%	1.2%	3.30 (1.55–7.02)**

***p<0.001, **p<0.01, *p<0.05

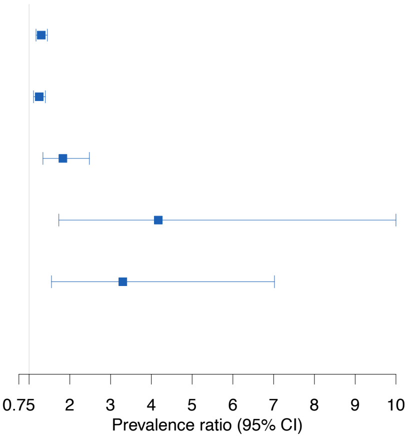

b) 31–45 years

Cardiometabolic condition	Non–ADHD (N=8069)	ADHD (N=890)	Prevalence ratio (95% CI)
Any	38.2%	45.2%	1.18 (1.10–1.28)***
Obesity	32.9%	38.2%	1.16 (1.06–1.27)**
Hypertension	11.0%	15.2%	1.39 (1.17–1.64)***
Type–2 diabetes	1.3%	2.8%	2.18 (1.42–3.35)***
Myocardial infarction	0.4%	1.2%	2.77 (1.42–5.42)**

***p<0.001, **p<0.01, *p<0.05

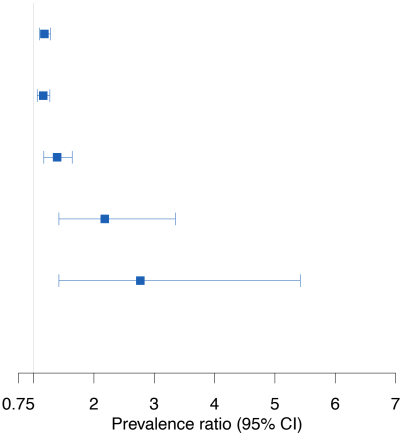

c) 46 years and older

Cardiometabolic condition	Non–ADHD (N=12855)	ADHD (N=583)	Prevalence ratio (95% CI)
Any	52.6%	57.5%	1.09 (1.02–1.18)*
Obesity	31.3%	37.6%	1.20 (1.08–1.34)***
Hypertension	36.0%	34.5%	0.96 (0.86–1.07)
Type–2 diabetes	4.8%	8.1%	1.69 (1.27–2.24)***
Myocardial infarction	2.1%	3.6%	1.70 (1.10–2.63)*

***p<0.001, **p<0.01, *p<0.05

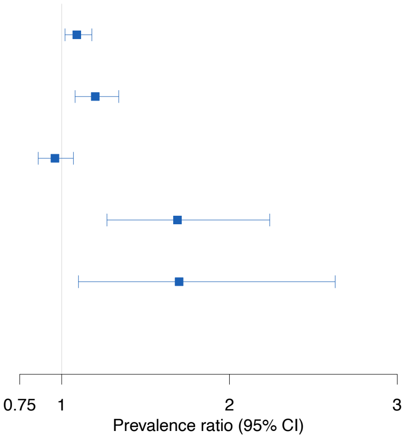

Supplement: Supplementary file 3 — Additional file 3: Figure S3. Prevalence and prevalence ratios of cardiometabolic conditions among women with and without ADHD stratified by age. Abbreviations: ADHD, attention-deficit/hyperactivity disorder; CI, confidence interval; CMC, cardiometabolic condition; GAD, generalized anxiety disorder; OCD, obsessive-compulsive disorder; PTSD, posttraumatic stress disorder; SUD, substance-use disorder. [file 12916_2023_3160_MOESM3_ESM.pdf]
