# Supplementary material for: Psychiatric comorbidities in women with cardiometabolic conditions with and without ADHD: a population-based study
Source: BMC Med. 2023 Nov 20;21:450. doi: 10.1186/s12916-023-03160-7 (PMC10659052; doi:10.1186/s12916-023-03160-7)

a) 18–30 years

Psychiatric disorder	CMC (N=1102)	CMC + ADHD (N=288)	Prevalence ratio (95% CI)
Any	50.1%	86.1%	1.72 (1.60–1.85)***
Depression	31.9%	61.8%	1.94 (1.71–2.19)***
Bipolar Disorder	2.5%	8.7%	3.54 (2.09–6.01)***
Social Phobia	19.7%	42.7%	2.17 (1.81–2.60)***
GAD	36.0%	53.8%	1.49 (1.31–1.71)***
PTSD	17.8%	42.0%	2.36 (1.96–2.85)***
OCD	6.8%	21.5%	3.16 (2.32–4.31)***
Alcoholism/SUD	2.0%	7.6%	3.83 (2.15–6.81)***
**Current psychiatric symptoms**			
Moderate/severe depressive	49.5%	71.9%	1.45 (1.32–1.59)***
Moderate/severe anxiety	35.5%	60.4%	1.70 (1.51–1.93)***
PTSD over cut–off	33.9%	60.8%	1.79 (1.58–2.03)***
**Type of self–harm**			
Self–harm without suicide intent	28.6%	33.7%	1.18 (0.98–1.42)
Self–harm with suicide intent	18.9%	35.1%	1.86 (1.52–2.27)***

***p<0.001, **p<0.01, *p<0.05

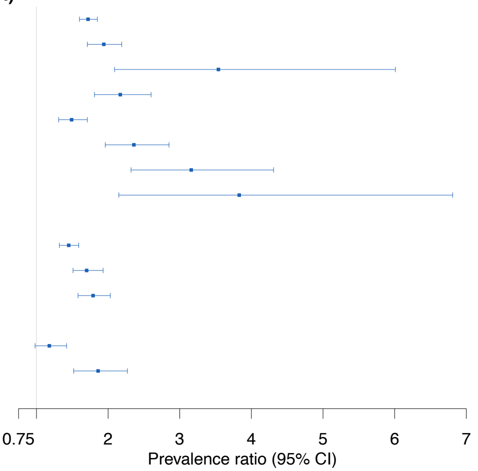

b) 31–45 years

Psychiatric disorder	CMC (N=3013)	CMC + ADHD (N=394)	Prevalence ratio (95% CI)
Any	40.6%	79.2%	1.95 (1.83–2.09)***
Depression	21.7%	50.5%	2.33 (2.07–2.62)***
Bipolar Disorder	2.0%	8.1%	4.08 (2.69–6.18)***
Social Phobia	11.1%	36.0%	3.26 (2.76–3.85)***
GAD	23.9%	53.6%	2.24 (2.01–2.51)***
PTSD	14.8%	39.6%	2.67 (2.30–3.10)***
OCD	3.5%	14.7%	4.26 (3.15–5.78)***
Alcoholism/SUD	3.4%	14.7%	4.39 (3.24–5.96)***
**Current psychiatric symptoms**			
Moderate/severe depressive	41.9%	70.3%	1.68 (1.56–1.81)***
Moderate/severe anxiety	29.0%	58.1%	2.01 (1.81–2.22)***
PTSD over cut–off	28.6%	60.4%	2.11 (1.91–2.33)***
**Type of self–harm**			
Self–harm without suicide intent	16.3%	25.6%	1.58 (1.31–1.90)***
Self–harm with suicide intent	8.2%	25.4%	3.08 (2.51–3.79)***

***p<0.001, **p<0.01, *p<0.05

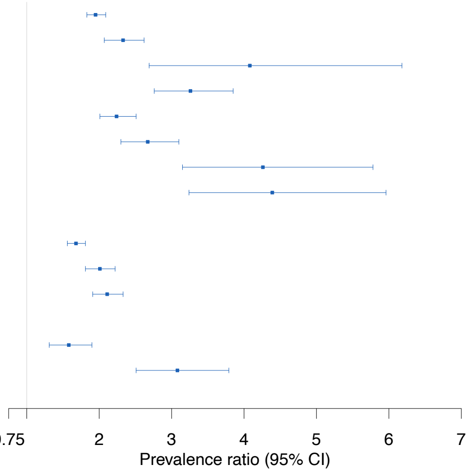

c) 46 years and older

Psychiatric disorder	CMC (N=6395)	CMC + ADHD (N=328)	Prevalence ratio (95% CI)
Any	24.8%	72.6%	2.92 (2.70–3.16)***
Depression	11.4%	42.7%	3.74 (3.24–4.31)***
Bipolar Disorder	1.3%	10.4%	8.18 (5.57–12.00)***
Social Phobia	5.5%	25.3%	4.57 (3.70–5.65)***
GAD	11.1%	38.4%	3.47 (2.98–4.05)***
PTSD	9.9%	37.8%	3.83 (3.27–4.48)***
OCD	0.9%	9.8%	11.30 (7.44–17.30)***
Alcoholism/SUD	3.2%	17.4%	5.42 (4.13–7.11)***
**Current psychiatric symptoms**			
Moderate/severe depressive	24.7%	62.5%	2.53 (2.31–2.78)***
Moderate/severe anxiety	14.5%	38.1%	2.63 (2.27–3.06)***
PTSD over cut–off	23.6%	50.6%	2.15 (1.91–2.41)***
**Type of self–harm**			
Self–harm without suicide intent	5.5%	14.0%	2.53 (1.90–3.37)***
Self–harm with suicide intent	2.7%	10.4%	3.90 (2.75–5.54)***

***p<0.001, **p<0.01, *p<0.05

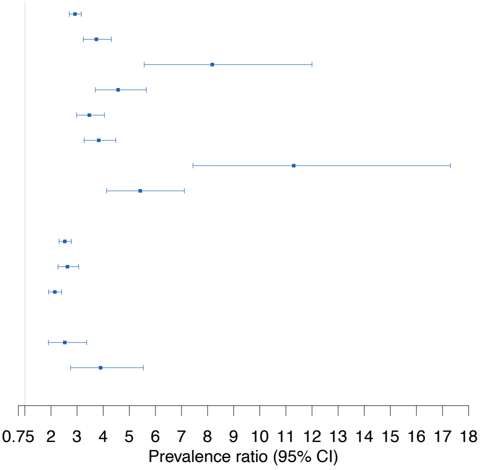

Supplement: Supplementary file 4 — Additional file 4: Figure S4. Prevalence and prevalence ratios of psychiatric disorders among women with cardiometabolic conditions and with/without co-occurring ADHD stratified by age. Abbreviations: ADHD, attention-deficit/hyperactivity disorder; CI, confidence interval; CMC, cardiometabolic condition; GAD, generalized anxiety disorder; OCD, obsessive-compulsive disorder; PTSD, posttraumatic stress disorder; SUD, substance-use disorder. [file 12916_2023_3160_MOESM4_ESM.pdf]
